# Tracheobronchopathia Osteochondroplastica: Two Cases Reports and Review of Literature

**DOI:** 10.1097/MD.0000000000003396

**Published:** 2016-05-13

**Authors:** Na Wang, Fei Long, Shujuan Jiang

**Affiliations:** From the Department of Pulmonary Medicine, Shandong Provincial Hospital Affiliated to Shandong University, Jinan, Shandong, China.

## Abstract

Tracheobronchopathia osteochondroplastica (TO) is a relatively rare and benign disease of unknown etiology that is characterized by the accumulation of diffuse cartilaginous and osseous nodules protruding into the anterolateral walls of the trachea and bronchus. However, TO is easy to ignore or misdiagnose due to its nonspecific clinical manifestation. A chest computed tomography (CT) scan with a fiber bronchoscope and pathological biopsy shows the clinical features supporting the ultimate diagnosis.

Here, we report 2 misdiagnosed cases of TO and review the literature to further define the diagnosis for clinicians. The first case was a 34-year-old male admitted to the hospital because of recurrent cough and intermittent fever for 10 years. CT scans showed irregular stenosis of the main bronchus and bronchofibroscope showed multiple nodules producing into the lumen. He was initially misdiagnosed of bronchial tuberculosis and received antitubercular agents for nearly half year. Symptoms got no relief and another bronchofibroscope with biopsy tests in our hospital exactly diagnosed of TO. Symptoms were significantly relieved after receiving budesonide associated with antibiotics, etc. Another case was a 46-year-old woman presenting with a history of repeated hoarseness for 8 years and a 2-month exacerbation. She underwent an electronic laryngoscopy 3 times and was diagnosed of laryngitis. Symptoms got no relief after antiinflammatory. CT scan indicated variable degrees of stenosis and calcification of the distal trachea and main bronchi and bronchofibroscope showed dozens of white nodules extruding into the lumen. Histopathologic findings revealed the ultimate diagnosis of TO and antiinflammatories, spasm relievers, and inhaled corticosteroids, showed apparent effects.

Poor specificity of TO is observed in clinical manifestation and laboratory inspection. However, a CT scan associated with a bronchoscopy and histopathologic examination greatly contributes to a definitive diagnosis. No specific treatments are recommended, except treatments to alleviate symptoms. Thus, it is of great importance to consider TO when facing unsolved respiratory or external respiratory symptoms to improve the quality of life.

## INTRODUCTION

Tracheobronchopathia osteochondroplastica (TO) is a rare disorder with a benign course. It is characterized by the accumulation of diffuse cartilaginous and osseous nodules protruding into the mucosa of the trachea and bronchus. These hallmarks are seen using a chest computed tomography (CT) scan and fiber bronchoscope. A pathological biopsy is indispensable for the final diagnosis. The incidence rate of TO is inconsistent with its hallmarks on imaging tests and endoscopy. It was first described in detail by Rokitansky and Wilms in the 19th century after an autopsy. As of June 2015, approximately 500 cases have been reported throughout the world. However, only 70 cases have been reported in China since 1991. Meyer et al^1^ reported that the incidence of TO was only 0.3% in autopsies and 1/125 to 1/5000 with bronchoscopy. The low incidence may be due to misdiagnosis, underreporting, and ignorance of the disease. Thus, considering TO when diagnosing an unsolved cough and chronic inflammatory patients is very valuable. Because of its obscure and debated etiology, adopting methods to prevent its progression and improve its diagnosis is necessary. Here, we report 2 cases of TO and summarize many facets of TO to improve its diagnosis.

## CASE REPORTS

### Case 1

**FIGURE 1:**
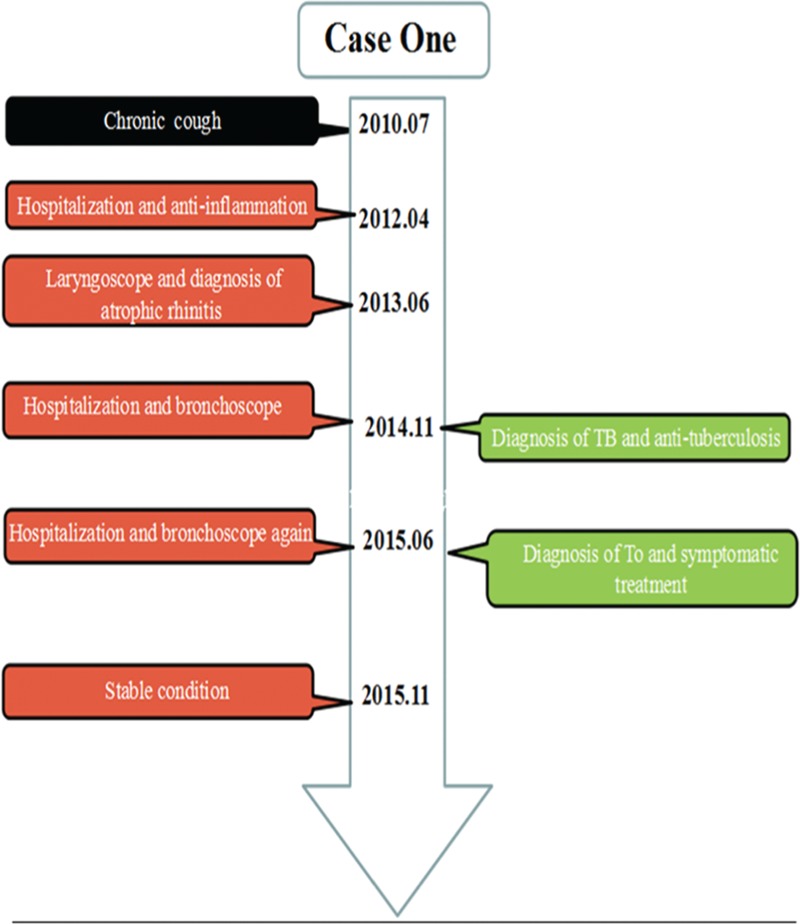
The timeline of interventions and outcomes for case 1.

**FIGURE 2:**
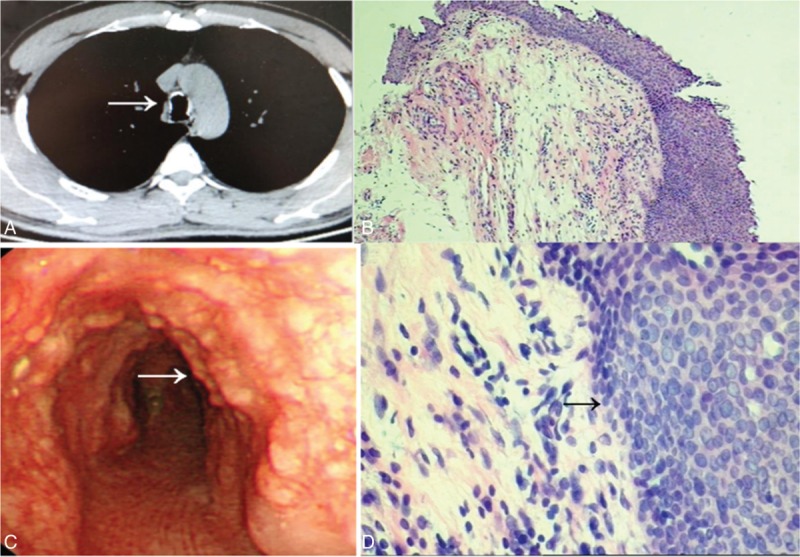
(A) CT scan of case 1 demonstrates irregularly thickened and abroad calcified nodules of the trachea. Arrow shows the calcific ring. (B) Bronchoscopy findings show multiple polypoid, white lesions covered with the anterior and lateral walls of trachea. Arrow shows the calcific nodules. (C and D) Histological appearance indicates infiltration of chronic inflammatory cells and arrow shows diffuse epithelial squamous metaplasia in submucosa (HE staining, original magnification ×100, ×400).

A 34-year-old man was admitted to a hospital for frequent episodes of cough and expectoration for the past 5 years. Due to continuous existence of symptoms and high lab values, he was misdiagnosed with pneumonia and received many antiinflammatory treatments, including amikacin, piperacilin, and suldactum in a local hospital. A bronchoscopy showed diffuse nodules invading the mucosa of the trachea. No evidence of *Mycobacterium tuberculosis* was found, and he was diagnosed of negatively bronchial tuberculosis and accepted antituberculosis treatments (Figure 1). The above symptoms worsened. To evaluate the nature of the lesions, he was admitted to the respiratory department of our hospital. He had a history of fracture of the left leg fibula due to trauma. Luckily, there were no sequelae. He drank about 100 ml of white wine daily and denied smoking. His life signs and physical examination were within normal limits, and no dry and wet rales were heard. Routine tests, including blood tests and liver and kidney functions were normal. A specific examination, including a tuberculosis antibody (TBAb) screen, yeast glucan, galactomannan test (GM test), γ-IFN for *M. tuberculosis* (T-spot) and a sputum screen for acid-fast bacilli were all normal. The erythrocyte sedimentation rate was 9 mm/h. The sputum culture grew *Klebsiella pneumoniae*. His spirometry was suggestive of high airway resistance, and the other indicators were normal. A thoracic CT scan revealed irregular stenosis of the left main bronchi accompanied by ample high density bone tissues (Figure 2A). A direct visualization using fiberbronchoscope showed diffuse white nodules protruding into the lumen, similar to cobblestone, sparing the posterior wall (Figure 2B). Due to the stiffness of the nodules, a forceps biopsy was difficult to perform. Histological appearance indicates infiltration of chronic inflammatory cells and diffuses epithelial squamous metaplasia in submucosa (Figure 2C and D). Symptoms were significantly relieved after receiving moxifloxacin, azithromycin, salbutamol, budesonide associated with an antitussive, expectorant, etc. The patient was required to follow-up in the outpatient department upon any deterioration.

### Case 2

**FIGURE 3:**
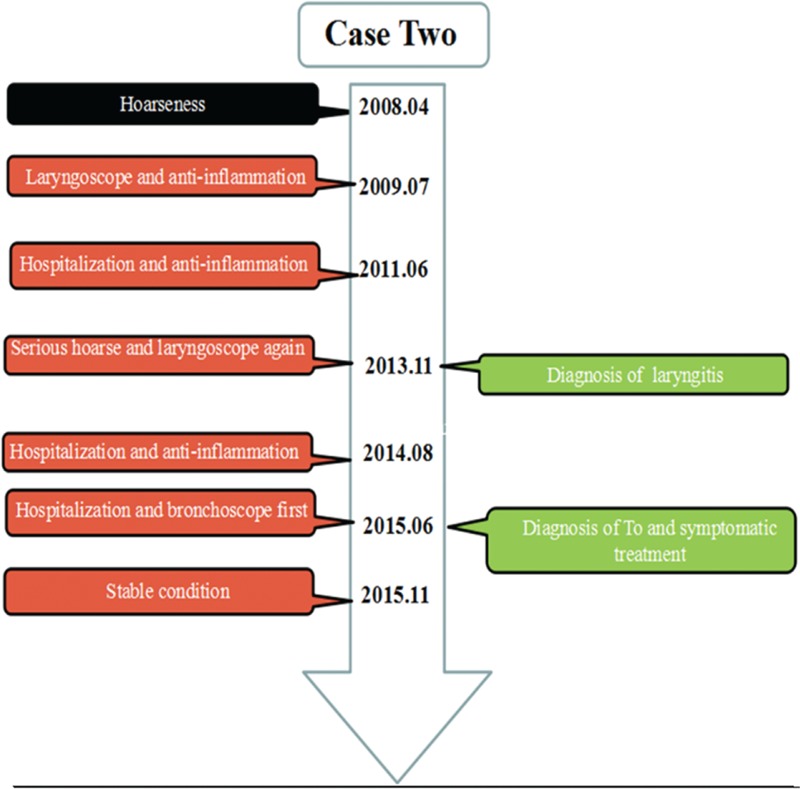
The timeline of interventions and outcomes for case 2.

**FIGURE 4:**
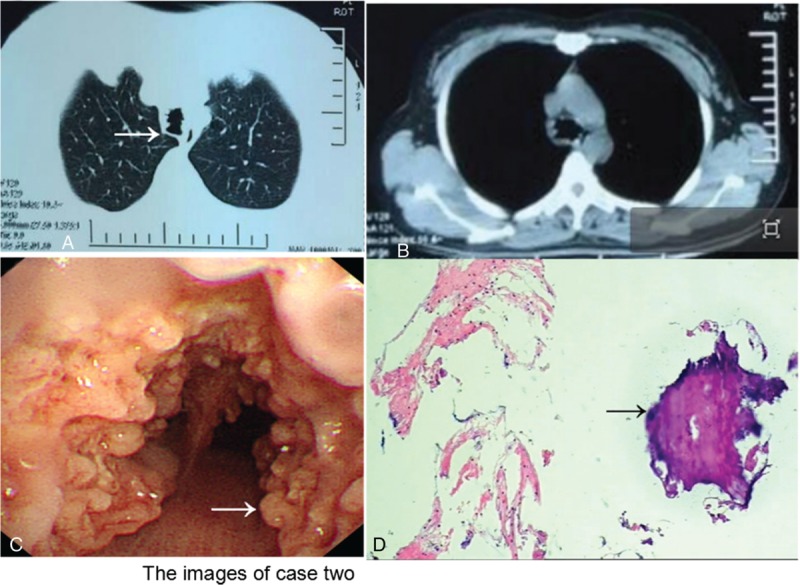
(A) Lung window CT of case 2 shows different degrees of luminal stenosis. (B) Mediastinal window CT of indicates calcified nodules protruding into lumen of trachea. (C) Widely small cobblestone nodular eminences distributing out of posterior wall are seen under bronchoscopy. Arrow shows the multiple nodules. (D) Histological examination appearances a central island of osteocytes apart from bronchial mucosa. Arrow shows the bone tissue (HE staining, original magnification ×100).

A 46-year-old woman was brought to the hospital with 8 years of progressive hoarseness and a 2-month exacerbation. To investigate the cause of repeated hoarse, the patient underwent an electronic laryngoscopy twice, which showed laryngitis. Antiinflammatory therapies relieved the symptoms to some extent. Two months ago, the patient was severely hoarse, including even aphonic. A third electronic laryngoscopy showed chorditis and an endotracheal tumor with bilateral vocal tract congestion, swelling, attaching albuginea, and mucosa erosion (Figure 3). The patient had a history of a panhysterectomy due to a hysteromyoma. She was a dove breeder for the latest 25 years and denied cigarette smoking and drinking. Abnormal changes were not found after a physical examination, except coarse breathing sounds and wet rales in the inferior lobe of the right lung. A blood test, tumor markers, sputum test for acid-fast bacilli, etc. were all normal, except for the liver function which showed ALT 65 U/L and globulin 19.6 g/L. Spirometry was suggestive of an obstructive ventilatory disturbance. A CT scan indicated variable degrees of stenosis and calcification of the distal trachea and main bronchi, accompanied by consolidation of the inferior lobe of the right lung (Figure 4A and B). A fiberbronchoscopy (FOB) showed signs of dozens of white nodules extruding into the lumen of trachea and bronchi and free from peripheral bronchial tree. Nodules like paving stones pervaded the wall, sparing the posterior aspects and possessing a rigid property (Figure 4C). Histological examination appearances a central island of osteocytes apart from bronchial mucosa (Figure 4D). Treatments, including antiinflammatories, spasm relievers, and inhaled corticosteroids, showed apparent effects. She was discharged from hospital on day 20.

## DISCUSSION

With the widespread use of bronchoscopies in the modern era, TO has become a relatively common disorder. However, because of misdiagnosis, underreporting and ignorance, the incidence of TO is only 0.3% at autopsies and 1/125 to 1/5000 with bronchoscopies.^1^ People above 50 years old are the victims of TO. Children and people younger than 50 years old rarely have TO. Simsek et al^2^ reported on a 9-year-old girl with TO. There is no sex predominance. Chroneou et al^3^ ever reported that men are more at-risk than women, which was consistent with our findings. We gathered 121 cases of TO patients, both domestic and international, and analyzed its incidence: men, 56.20% (68/121) and women, 43.80% (53/121). The youngest was 9 years old and the oldest 85 years old. However, Leske et al^4^ had the opposite conclusion. They noted that the recruitment method was dedicated to female predominance, similar to how atrophic rhinitis at one time appeared more in woman. A clear geographical link was obscure. Atypical features provides no help in the prevention of TO. Determining the potential factors and causes remains critical.

There was no consensus on etiology. Several theories addressed the occurrence and development of TO. Zack and Rozenshtein^5^ noted that undifferentiated connective tissue cells situated in the submucosal and elasticity layer transformed to cartilage and bone. Smid et al^6^ indicated that undifferentiated, pluripotent embryonic cells could differentiate to osteocytes and chondrocytes, even in bone marrow under chemical or hormonal stimulation. Tajima et al^7^ found that bone morphogenetic protein 2 (BMP-2) cooperated with transforming growth factor-β1 (TGF-β_1_) and promoted the formation of calcific foci. The participants provide written informed consent to participate. The subjects’ rights and interests are protected well in the whole process. The research hereby is approved by the Medical Ethics Committee of Shandong Provincial Hospital affiliated to Shandong University. Our first case was an electric welder and the second was a dove breeder for the past 25 years. The 2 patients have potential risks due to occupational exposure, including chemical substances and proteins in avian droppings. No evidence or relative findings showed any physical–chemical factors attributed to tracheal ossification or Ca^2+^/P^3+^ deposition. While hypersensitivity pneumonitis, caused by inhalation of a wide variety of offending antigens, can also lead to respiratory symptoms, all 2 patients’ CT scan showed no classical diffuse ground glass opacity admixed with multiple nodules and none of classic histologic triad including interstitial infiltrate, cellular bronchiolitis, and poorly formed granulomas were found. No use of systematic corticosteriods got symptoms relief. In light of these, hypersensitivity pneumonitis was not taken consideration.

Because of its unspecific manifestation, most cases are incidentally found at autopsy. Few patients had problems with intubation when undergoing surgery.^8,9^ From the 121 cases we gathered, the symptom frequencies were cough (90.0%), hemoptysis (44.6%), dyspnea (35.5%), chest pain (19.8%), and hoarseness (5.8%), which were consistent with Lundgren and Stjernberg.^10^ Our first case had an unsolved cough and intermittent fever with the highest temperature being 39.6°C. Thus, he was given the primary diagnosis of a pulmonary infection. Either the airway infection accelerated TO or the damage caused by TO on the local structure of wall caused a stubborn infection, although the definitive relationship was unresolved. The second case had chronic hoarseness for 8 years and respiratory symptoms evidenced vocal infringement under laryngeal endoscopy.

TO is usually combined with other disorders. Jabbardarjani et al^11^ reviewed 10 cases with accompanying tuberculosis, atrophic rhinitis, and anthracosilicosis. Other researchers have found TO patients with involved in silicosis, lung cancer, and non-Hodgkin lymphoma.^12–14^ Asthma, atrophic rhinitis, and acromegaly have explicit relationships with TO and amyloidosis, relapsing polychondritis, and tracheobronchomalacia.^4,10^ Our first case complained of rhinobyon and dysosmia. A nasal endoscopy showed diffusely atrophic nasal mucosa. Lastly, he was diagnosed with atrophic rhinitis in 2013. Yokoyama et al^15^ illustrated 19% of patients with malignant adenocarcinoma also had TO, which is a high incidence rate. The second case had a misdiagnosed extratracheal tumor under the protruding nodules below the vocal cord before the specific diagnosis of TO was made. Sometimes TO can be involved in the cardiovascular system. Muller et al^16^ and Vidal et al^17^ separately reported TO patients with endocardial calcification, which was bad for the progression of TO in cases of acute cardiovascular disease events.

A routine investigation, especially imaging examinations, for TO diagnosis is indispensable. An X-Ray is less sensitive for calcific lesions than a CT scan. A CT scan is an important noninvasive test to detect lesions 3 mm or smaller. Lesions are generally 2 to 5 mm. Danckers et al^18^ first reported a TO subjected with a 2.8 cm × 2.0 cm × 4.0 cm mass, which causing obstructive atelectasis and acute dyspnea. A classical manifestation shows diffuse osseous deposits protruding into the anterior and lateral lumen from the main bronchus to the segmental bronchus. They rarely involve the vocal cords and bronchiole terminals. If erosion is involved in posterior wall, frequently amyloidosis, tracheobronchomalacia, bronchial sarcoma are considered. A CT of our 2 cases corresponded with the above description.

With the rapid development of bronchoscopy, the rate of diagnosis has improved to 0.7%. Nearly all the patients were diagnosed by bronchoscopy, which is able to show the clinical hallmarks.^19^ Our 2 cases all showed multiple white nodular lesions with a hard texture in the anterolateral wall and looked like paving stone from the bronchus to the distal airway. Besides few patients appear to have atelectasis because of the massive nodules, though the mucosa was swollen with lesions blocking the lumen.^20,21^ The histopathological examination in the first case indicated epithelial squamous metaplasia, formation of cartilage and bone, and existence in the bone marrow. These changes, especially the squamous metaplasia of the epithelium and loss of normal airway architecture, are likely to affect the defense mechanisms, especially mucociliary clearance, leading to chronic infection of respiratory syetem.^4^ The indispensability of bronchoscopy in the diagnosis remains uncertain. Because many nodular diseases, such as amyloidosis, relapsing polychondritis, and tracheobronchomalacia, mimic the TO hallmarks, a histopathological examination is the only way to ensure a proper diagnosis. It remains unclear if pathogens promote the development of TO and K. Ozaenae could damage the mucociliary protective mechanism and simultaneously facilitate atrophic rhinitis.^22^ The first case was diagnosed as atrophic rhinitis. However, a subspecies of pneumoniae rather than K. Ozaenae was cultured. None of the literature has reported a pneumoniae subspecies to be associated with TO. The patient was given cefoperazone/sulbactam 3.0 g twice a day and a week later, the symptoms obviously relieved.

A spirometry test showed no apparent changes. Our first case had increased airway resistance. The second case showed moderately obstructed ventilation. Lundgren and Stjernberg^10^ reported that 8 out of 9 patients presented with obstructed ventilation and low inspiration and expiration levels in a flow-volume loop. Spirometry tests can predict the progression of TO.

A clinician should be aware of TO when diagnosing an unsolved cough, recurrent infection, and hoarseness. Swollen mucosa and diffuse nodules coating the wall from the principal bronchus to the segmental bronchus are also features of amyloidosis, tracheobronchomalacia, bronchial sarcoma, and differential diagnosis is essential. The relative differential diagnoses are shown below. Bronchial tuberculosis violating the mucosal lumen, possessing narrowed, wavy, straight shape, and severely combined with hilum of lung and mediastinal lymphadenectasis. In the early stages, the mucous membrane is characterized by chronic inflammation. Terminally, the mucosal membrane develops caseous necrosis or cicatricial stenosis of the lumen. It is of great significance that regular antituberculosis treatment is effective. A CT of tracheal amyloidosis shows limited or diffuse nodules protruding into lumen and located mostly in the posterior wall. Most importantly, biopsy specimens are positive for Congo red staining. The liver and kidneys can also be involved. Relapsing polychondritis appears as chondral calcification in addition to chronic sinusitis, inner ear damage, and erosive arthritis. Luckily, glucocorticoids with or without immunosuppressants rapidly modify the disease course. The first case was misdiagnosed with bronchial tuberculosis and received antituberculosis treatment for half a year before being admitted to our hospital, which led to great damage to the patient's liver function. Clearly, decreasing the frequency of misdiagnoses is critical.

TO has benign transformation potential and rarely needs specific treatment. Mathlouthi et al^23^ and van Nierop et al^24^ reported that patients in a stable condition were followed for 15 and 25 years. Most inpatients receive palliative treatments, such as antiinflammatories and antitussives. When a patient presents with acute breathlessness, inhalation of beclometasone dipropionate and budesonide can quickly ease his or her symptoms.^8^ If a patient's condition becomes more severe and is complicated by severe airway stenosis or recurrent obstructive infection, laser ablation, surgical resection, and even radiotherapy are suggested. Bronchoscopy laser vaporization is the most exact curative treatment.^24^ Our 2 cases were successfully discharged after receiving antibiotics, glucocorticoids, etc.

In conclusion, TO has benign potential. Poor specificity is observed in clinical manifestation and laboratory inspection. However, a CT scan can show central airway mucosal irregularities and calcification. A bronchoscopy and histopathologic examination greatly contribute to a definitive diagnosis. No specific treatments are recommended, except treatments to alleviate symptoms. Sometimes a therapeutic endoscopy or tracheotomy is used to treat severe dyspnea, thereby improving the quality of life.
